# The Kyoto Prognostic Index for patients with diffuse large B-cell lymphoma in the rituximab era

**DOI:** 10.1038/bcj.2015.111

**Published:** 2016-01-15

**Authors:** T Kobayashi, J Kuroda, I Yokota, K Tanba, T Fujino, S Kuwahara, R Isa, J Yamaguchi, E Kawata, T Akaogi, H Uchiyama, H Kaneko, N Uoshima, Y Kobayashi, S Teramukai, M Taniwaki

**Affiliations:** 1 Division of Hematology and Oncology, Department of Medicine, Kyoto Prefectural University of Medicine, Kyoto, Japan; 2 Department of Biostatistics, Graduate School of Medical Science, Kyoto Prefectural University of Medicine, Kyoto, Japan; 3 Department of Hematology, Japanese Red Cross Kyoto Daini Hospital, Kyoto, Japan; 4 Department of Hematology, Japanese Red Cross Kyoto Daiichi Hospital, Kyoto, Japan; 5 Department of Hematology, Aiseikai Yamashina Hospital, Kyoto, Japan

The International Prognostic Index (IPI; age, the disease stage according to the Ann Arbor system, serum lactate dehydrogenase (LDH), Eastern Cooperative Oncology Group (ECOG) performance status (PS) and the presence of extranodal involvement (ENI)) has been the traditionally utilized prognostic tool for diffuse large B-cell lymphoma (DLBCL) treated with cyclophosphamide, doxorubicin, vincristine, and prednisolone (CHOP) or CHOP-like chemotherapy.^1^ Although the incorporation of rituximab (Rit) into CHOP has markedly improved the treatment outcome of DLBCL, this resulted in the reduction of the prognostic impact of the IPI. Even with the alternative revised IPI (R-IPI), it also failed to accurately identify the small proportion of patients at risk for a short survival period. Indeed, in the R-IPI-defined poor-risk group, ~40% of the patients survived for a relatively short period within 2 years, whereas more than half were cured by R-CHOP, indicating that the sensitivity of R-IPI-defined poor-risk group for identifying the potential short survivors was <50%.^2^ More recently, the National Comprehensive Cancer Network (NCCN)-IPI has been proposed for DLBCL that emphasizes the prognostic values of age, high-serum LDH and the specific sites of ENI.^3^ However, the NCCN-IPI was again not sufficient for discriminating patients at risk for very short survival, and this was also the case with other indexes, such as the modified Glasgow Prognostic Score (mGPS) consisting of serum C-reactive protein (CRP) and albumin (ALB) levels for DLBCL.^4, 5^ In addition, although informative, the prognostic prediction of DLBCL on the basis of biological features using either an immunohistochemical method or gene expression profiling remains difficult to be generally adapted in daily clinical practice.^6, 7^

We here tried to generate a new prognostic index that is easy to use in daily clinical practice and more accurately predicts the outcome of DLBCL, especially that of the small proportion of ultrahigh-risk patients in the Rit era. We retrospectively analyzed the clinical records of 465 patients with histologic diagnosis of DLBCL who were treated at three independent institutes from January 2006 to April 2014. In general, patients were treated with three courses of R-CHOP or R-CHOP-like therapy followed by involved-field radiation for localized disease and six to eight courses of R-CHOP or R-CHOP-like therapy for advanced disease. Minute adjustment of a therapeutic regimen was allowed at the doctor's discretion. Patients were excluded from the analysis if they were HIV positive, were complicated with other hematological diseases, transformed DLBCL, primary central nervous system (CNS) lymphoma or had a major coincident illness that precluded an attempt at curative treatments. This study was conducted in accordance with the ethical principles of the Declaration of Helsinki, and was approved by the institutional review boards. The methods for the statistical analyses were described in Supplementary Information.

We randomly selected 323 patients (70% of all patients) as a training cohort to identify prognostic factors for building up a new prognostic model, and selected the remaining 142 patients (30%) as a validation cohort. There were no significant differences in patients' characteristics between the training and the validation cohorts (Supplementary Table S1). The median overall survival (OS) and progression-free survival (PFS) of all patients were not reached during the median follow-up of 32.2 months, and the estimated 3-year OS and PFS were 78.5 and 67.4%, respectively (Figures 1a and b). Among 14 extranodal sites: liver/gastrointestinal tract (*n*=77), bone marrow (BM; *n*=37), lung/pleura (*n*=34), bone (*n*=25), head and neck (*n*=17), genitourinary tract (*n*=16), testis (*n*=14), breast (*n*=14), spleen (*n*=13), CNS not as the primary site (*n*=10), adrenal gland (*n*=10), skin (*n*=9), thyroid (*n*=7) and peripheral blood (*n*=4), ENI in the BM (*P*=0.002), bone (*P*=0.028), skin (*P*<0.001) or lung/pleura (*P*=0.002) at diagnosis was statistically significantly associated with poorer OS by the univariate analysis (Supplementary Figure S1). Accordingly, we evaluated age 60 years and older, serum LDH ratio (>1–3 or ⩾3), Ann Arbor stage III–IV, ECOG-PS (⩾2), ENI (BM, bone, skin and/or lung/pleura), elevated serum CRP level (>1.0 mg/dl) and hypoalbuminemia (<3.5 mg/dl) as candidates for prognostic variable. Although all factors were significantly related to OS based on the univariate analysis, the prognostic factors that remained significant were LDH, PS, ALB and ENI based on the multivariate analysis in the training sample (Table 1). The weights of the variables were decided based on the estimated regression coefficients, and we derived the final prognostic index consisting of four factors, that is, LDH (>1–3, score 1; ⩾3, score 2), ECOG-PS (⩾2, score 1), ALB (<3.5 mg/dl, score 1) and ENI (BM, bone, skin and/or lung/pleura; score 1). When classified into four statistically significantly distinct risk groups in our new prognostic index, designated as the Kyoto Prognostic Index (KPI): low-risk group (L: score 0), low-intermediate risk (LI: score 1–2), high-intermediate risk (HI: score 3) and high risk (H: score 4–5), the 3-year OS rates were 96.4% for L, 84.7% for LI, 63.8% for HI and 33.3% for H (Figure 1c). The KPI was also predictive for PFS (3-year PFS: 84.4% in L, 70.2% in LI, 53.4% in HI and 24.1% in H; Figure 1d). Also in the validation cohort, the KPI was highly predictive for OS of DLBCL, demonstrating the statistically significant differences of 3-year OS rates among the four risk groups (Figure 1e). In addition, the KPI was also useful for the prediction of PFS in the validation cohort (Figure 1f).

Finally, we compared the predictive power of the KPI with that of the R-IPI and the NCCN-IPI in our cohort by examining the *c*-index and the relative Brier score reduction (RBSR) in the validation cohort. Although the *c*-indices of OS and PFS as determined by the R-IPI were 0.642 and 0.668, and those as determined by the NCCN-IPI were 0.736 and 0.749, the OS and PFS by the KPI were well correlated with *c*-indices of 0.740 and 0.703, respectively, indicating the model with the favorable capability for distinguishing the survival periods. The RBSR of OS and PFS by the KPI were 30.5 and 18.3%, compared with that determined by the R-IPI were 13.5 and 12.2%, and those as determined by the NCCN-IPI were 25.1 and 17.2%. These suggest that the KPI has a relatively greater ability for accurate survival prediction compared with the R-IPI and the NCCN-IPI. Indeed, the absolute differences in OS between the low- and high-risk groups were 85.7% by the KPI model compared with 42.2% by the R-IPI model and 62.0% by the NCCN-IPI model in the validation cohort (Figure 1e and Supplementary Figure S2). The greater capability of the KPI to identify the extremely poor prognostic group was also supported by the greater difference in the RBSR between the KPI and the NCCN-IPI rather than the difference in the *c*-index. By contrast, mGPS was not relevant at least in our cohort (data not shown).

Unlike both the R-IPI and the NCCN-IPI, our study demonstrated that older age had an insignificant impact on the outcome of DLBCL. Conceivably, the relatively higher proportion of the patients aged >60 (~80%) in our cohort diminished the negative impact of the older age in our study. In contrast, as has been reported,^4, 8, 9, 10^ the serum ALB level was identified as an independent prognostic factor for DLBCL in our study, although it was not included in the R-IPI or NCCN-IPI. Another change in the KPI was related to the extranodal sites at diagnosis, that is, BM, bone, skin and/or lung/pleura. This was consistent with the previous study which suggested that some specific extranodal sites, such as BM and pleura, seem to be more important for predicting the outcome for DLBCL than that of the number of extranodal lesions.^11^ Among R-IPI/NCCN-IPI-defined poor-risk groups, where about 40% of the patients were cured, there also remained a certain proportion of patients with extremely poor prognosis who did not respond to R-CHOP (-like) chemotherapies. It is very important that a new methodology be established that has increased sensitivity for the identification of those patients with a poor prognosis, because the R-IPI and the NCCN-IPI have only about 60% sensitivity for this identification. In this regard, the proposed KPI, a robust and feasible prognostic model, may provide a higher capability to discriminate especially the high-risk patients among newly diagnosed DLBCL. For those patients with more aggressive disease, other more intensive regimens rather than R-CHOP (-like) regimens, might be preferable as the first-line treatment.^12, 13, 14^

## Figures and Tables

**Figure 1:**
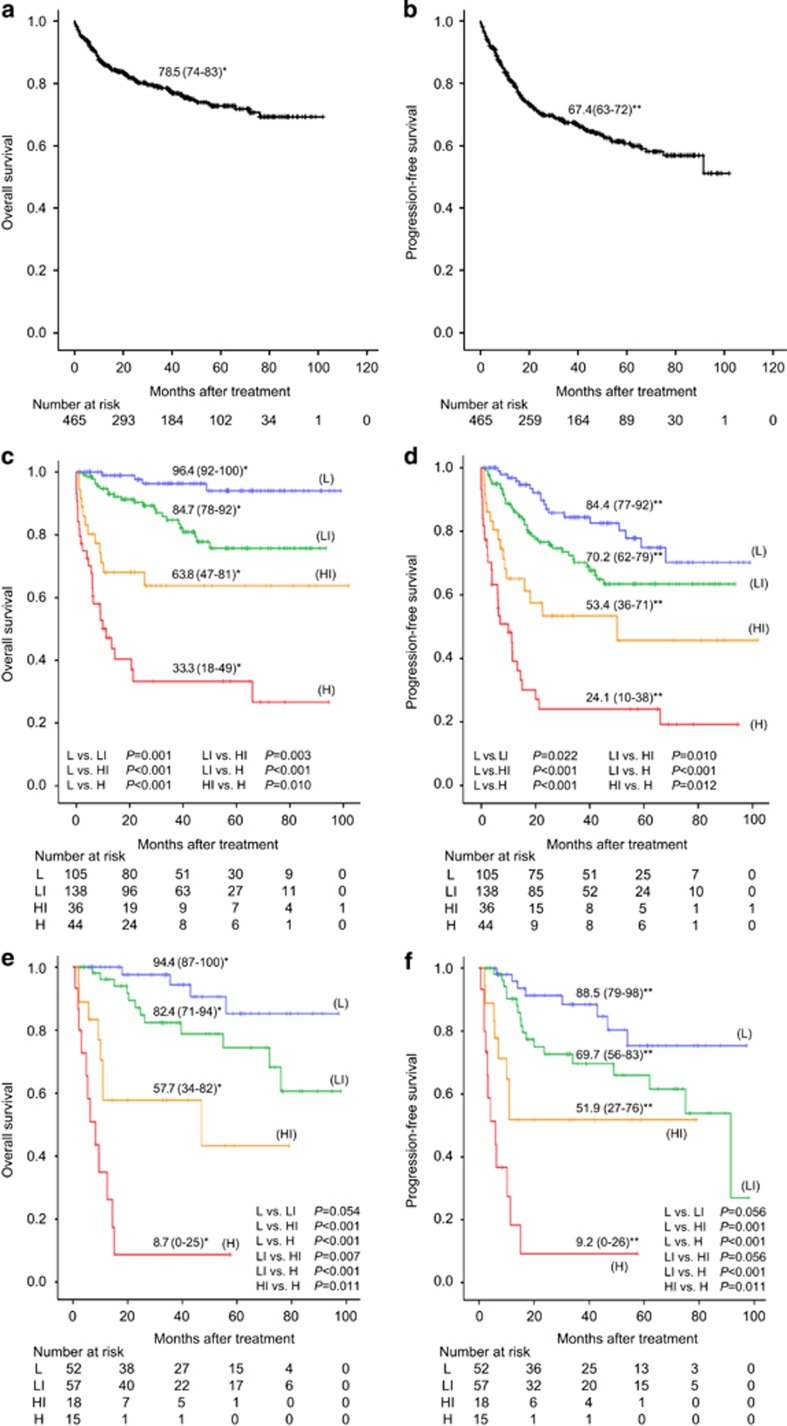
(**a**, **b**) OS (**a**) and PFS (**b**) of all 465-DLBCL patients analyzed with the Kaplan–Meier method. (**c**, **d**) OS (**c**) and PFS (**d**) according to the KPI in the training cohort. (**e**, **f**) OS (**e**) and PFS (**f**) according to the KPI in the validation cohort. *3-year OS (95% CI; %), **3-year PFS (95% CI; %).

**Table 1:** Statistical analyses of the prognostic variables for OS in the training cohort

*Baseline variable*	*HR*	*95% CI*	P-*value*
*Univariate Cox regression analyses for OS*			
Age >60 years	2.109	1.043–4.265	0.038
LDH ⩽1 × ULN	1	—	—
LDH >1 × ULN, ⩽3 × ULN	4.339	2.205–8.539	<0.001
LDH >3 × ULN	12.257	5.752–26.118	<0.001
Ann Arbor stage III–IV	3.13	1.779–5.507	<0.001
ECOG-PS ⩾2	6.498	3.958–10.670	<0.001
CRP >1.0 mg/dl	4.336	2.605–7.217	<0.001
ALB <3.5 mg/dl	5.592	3.273–9.554	<0.001
Extranodal disease^a^	3.324	2.048–5.397	<0.001

*Multivariate Cox regression analyses of variables for OS selected by backward stepwise regression*			
LDH ⩽1 × ULN	1	—	—
LDH >1 × ULN, ⩽3 × ULN	2.472	1.203–5.078	0.014
LDH >3 × ULN	3.688	1.571–8.657	0.003
ECOG-PS ⩾2	2.496	1.401–4.448	0.002
ALB <3.5 mg/dl	2.523	1.358–4.688	0.003
Extranodal disease^a^	1.713	1.031–2.844	0.038

Abbreviations: ALB, albumin; CI, confidence interval; CRP, C-reactive protein; ECOG, Eastern Cooperative Oncology Group; HR, hazard ratio; LDH, lactate dehydrogenase; OS, overall survival; PS, performance status; ULN, upper limit of normal range.

a Lymphoma involvement in the bone marrow, bone, skin or lung/pleura.
