# Supplementary material for: The Kyoto Prognostic Index for patients with diffuse large B-cell lymphoma in the rituximab era
Source: Blood Cancer J. 2016 Jan 15;6(1):e383–. doi: 10.1038/bcj.2015.111 (PMC4742628; doi:10.1038/bcj.2015.111)
Supplement: Supplementary Information [file bcj2015111x1.docx]

**Supplementary Information**

***Statistical analysis***

PFS was defined as the time from initiation of treatment to documented disease progression, or the date of death from any cause, whichever occurred first. Observations were censored on the date of death for patients dying as a result of causes unrelated to either lymphoma or treatment. OS was calculated from initiation of treatment to death from any cause. OS and PFS curves were estimated using the Kaplan-Meier method. The log-rank test was performed to compare curves. Univariate Cox proportional-hazards regression analyses were performed to evaluate the prognostic values of each variable. A new prognostic index to predict OS was identified using the multivariate Cox proportional-hazards regression model with backward elimination methods, with *p* < 0.05 considered as statistically significant. This study included 5 variables used for the classical IPI and the NCCN-IPI, namely, age, serum LDH level, Ann Arbor stage, ECOG PS, sites of extranodal involvement, and factors utilized in the mGPS, namely, serum CRP and albumin level. The Mann-Whitney U-test and the chi-square test were used to compare baseline characteristics between the training sample and the validation sample. We selected the best risk classification in an attempt to separate the prognosis of patients based on Akaike’s information criterion. The predictive ability of the proposed model was evaluated with the c-index and relative Brier score reduction (RBSR). The c-index is the conditional probability that the patients with a longer event time were estimated at a lower risk, given any pair with different event times, and was utilized for the measurement of separation of survival distributions among different risk groups. RBSR is the relative reduction in the Brier score that is the mean squared prediction error of the proposed model against the null model. The confidence interval (CI) was 95% for all analyses, and *p* < 0.05 was considered statistically significant. Statistical analyses were performed using SPSS (version 22; IBM, Armonk, NY, USA) and R 3.1.3 with the survC1 and ipred package.
