# Supplementary figures and images for: The Kyoto Prognostic Index for patients with diffuse large B-cell lymphoma in the rituximab era
Source: Blood Cancer J. 2016 Jan 15;6(1):e383–. doi: 10.1038/bcj.2015.111 (PMC4742628; doi:10.1038/bcj.2015.111)

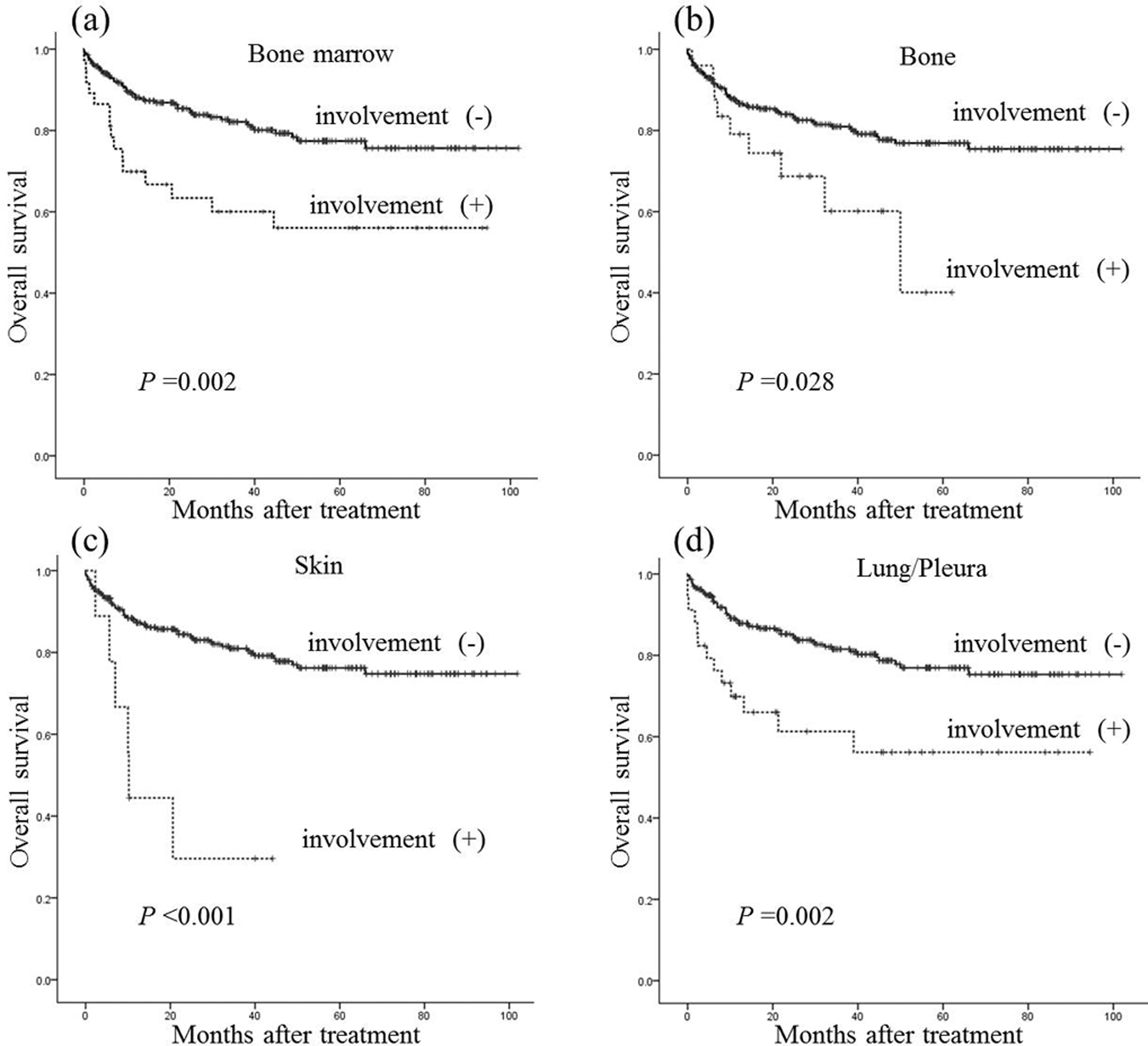

Supplement: Supplementary Figure S1 [file bcj2015111x2.tif]

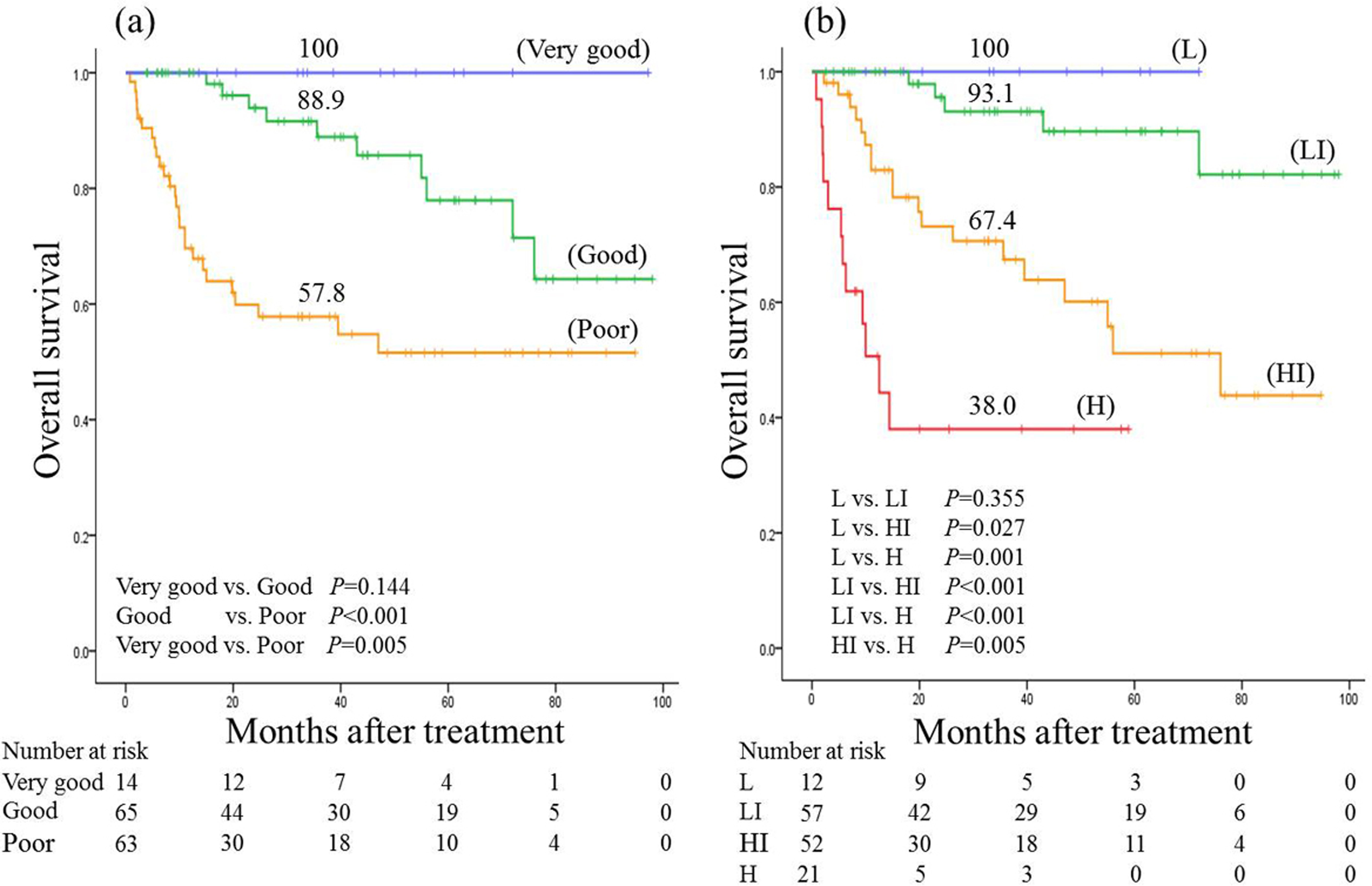

Supplement: Supplementary Figure S2 [file bcj2015111x3.tif]
